# Cancer Clinical Trial Patients’ Perceptions of Reporting Adverse Events Via an Electronic Platform

**DOI:** 10.1007/s43441-025-00804-z

**Published:** 2025-05-23

**Authors:** Minna Grahvendy, Bena Brown, Laurelie R. Wishart

**Affiliations:** 1 Cancer Trials Unit, Princess Alexandra Hospital, Queensland Health, Brisbane, QLD Australia; 2 School of Health and Rehabilitation Sciences, The University of Queensland, Brisbane, QLD Australia; 3 Southern Queensland Centre of Excellence in Aboriginal and Torres Strait Islander Primary Health Care, Metro South Hospital and Health Service, Brisbane, QLD Australia; 4 School of Public Health, The University of Queensland, Brisbane, QLD Australia; 5 Centre for Functioning and Health Research, Metro South Hospital and Health Service, Brisbane, QLD Australia; 6 Centre for Applied Health Economics, School of Medicine and Dentistry, Griffith University, Brisbane, QLD Australia; 7 Metro North Hospital and Health Service, Royal Brisbane and Women’s Hospital, Brisbane, QLD Australia

**Keywords:** Clinical trial, Patient-reported, Adverse event, CTCAE, PRO-CTCAE, PRO

## Abstract

**Background:**

Accurate adverse event (AE) reporting is critical to the success of clinical trials; over the last 10 years, momentum has been building to collect AE data directly from the patient. In this study, we investigated the use of an electronic, web-based platform by which cancer clinical trial patients self-reported AE data. In this report, we explored the perceptions and experiences of participants using the platform during participation in an interventional clinical trial.

**Methodology:**

Patients consenting to an interventional clinical trial run by a cancer clinical trial unit at a tertiary hospital in Australia were eligible for enrolment. Participants used an online platform to report symptomatic data weekly over 26 weeks. Participant feedback on the platform was collected via an implementation survey at baseline, week 12, and week 26 and a qualitative interview at week 26.

**Results:**

Participants agreed that the platform was acceptable, appropriate, and feasible in its purpose. This agreement remained throughout their participation on the study. Qualitative analysis of interview data revealed three major themes: accessibility and useability, platform functionality, and personal attributes and benefits/burdens. Participants reported areas for improvement, primarily around platform functionality and consideration of symptom burden limiting participant capacity to engage with the platform.

**Conclusions:**

To our knowledge, this is the first study that investigates, in-depth, the participant experience of self-reporting cancer clinical trial AE data via an electronic platform. Our study lends evidence from the participant-perspective to previously-reported studies investigating the feasibility of collecting AE data directly from the patient. Participants agreed that the platform was feasible and would be of benefit to future cancer clinical trial participants.

## Introduction

Accurate and complete adverse event (AE) data collection is vital to patient safety in cancer clinical trials. It is a standard requirement in cancer clinical trials for all AEs, whether related to intervention or not, to be assessed by the study site team and reported to the sponsor of the study in accordance with the study protocol [1]. Standardisation of AE reporting occurred in 1982 when the National Cancer Institute published the Common Toxicity Criteria, which allowed for assessment of AEs on a 5-point grading scale. Today, now known as the Common Terminology Criteria for Adverse Events (CTCAE), the tool incorporates terms for 837 AEs including laboratory findings, observable and measurable events, and symptomatic events [2].

Over the last ten years, work has focussed on the potential of collecting AE data directly from the patient. Wernicke et al. [3] compared spontaneous reporting with solicited reporting where AE data was collected from patients using a structured questionnaire; the solicited reporting resulted in more patients reporting more AEs. Multiple studies have shown that it is feasible to collect AE data directly from patients in the cancer clinical trial setting [4–6]. In line with this work, the National Cancer Institute has developed the patient-reported outcomes version of the CTCAE (PRO-CTCAE) [7]. The PRO-CTCAE is comprised of plain language terms for the approximately 10% of items in the CTCAE that correspond to symptomatic AEs and is designed for direct patient-reporting of symptomatic AEs. It provides patients with the ability to rate symptoms on attributes of presence/absence, amount, frequency, severity, and interference with daily life. A 5-point verbal descriptor scale is used to rate each attribute apart from the binary presence/absence assessment [8].

Despite this early feasibility data, very few studies have reported on the consumer experience of reporting adverse events using a patient-reported outcome instrument. Basch et al. [5] and Basch et al. [6] reported on an electronic version of the PRO-CTCAE administered to cancer clinical trial patients via tablet computer at clinic visits, however, their reporting on the patient experience in these studies is quite limited. They reported that patients provided feedback on the instrument via survey and shared that they found the instrument easy to understand, easy to use, and completing the instrument improved discussions with their treating clinical team [5, 6]. Liu et al. [9] reported on a mobile phone app designed to report blood pressure and diarrhoea for patients on a clinical trial receiving olaparib and cediranib for ovarian cancer. Feedback on the instrument was collected via phone interview and survey after one week and one month of app use respectively. Interview and survey data were consistent and showed that patients found the app easy to use and resulted in patients feeling more involved in their care and more connected with their treating team. Some negative feedback was reported including some difficulty in use of the app in understanding blood pressure measurements, and confusion around the meaning of 24-hour recall in relation to reporting diarrhoea.

In the current study, we investigated the use of an electronic, web-based platform by which cancer clinical trial patients self-reported AE data to the research team. Acknowledging the importance of the consumer experience in the viability of such tools, we specifically explored the perceptions and experiences of patients using the platform to report their symptomatic AEs during participation in an interventional cancer clinical trial.

## Materials and Methods

### Participants and Study Design

Patients consenting to an interventional clinical trial run by a cancer clinical trial unit at a tertiary hospital located in Australia were eligible for enrolment to this prospective, single-site study. Flyers advertising the study were distributed in the cancer outpatient clinics to medical oncology and haematology patients. Patients were eligible if they were newly enrolled into a clinical trial, had not yet started treatment on trial, and had access to internet and a personal electronic device. Following consent, participants were followed on study for a period of 26 weeks, or until they discontinued treatment on the clinical trial or voluntarily withdrew from the study or clinical trial.

The current study was approved by the Queensland Health Metro South Hospital and Health Service human research ethics committee (HREC/2021/QMS/73409) and all patients provided written, informed consent to participate in the study.

### Electronic Platform, Survey, and Administration

The electronic platform used in this study was the My Health My Way (MHMW; previously ScreenIT) platform developed by researchers at the hospital where this study took place [10]. MHMW is designed as a person-centred, web-based, electronic platform to capture common side effects experienced by patients with cancer, as well as data on physical, functional, and psychosocial factors [11]. The performance of the MHMW platform has been assessed in routine care within the head and neck cancer patient population at the hospital, and it has been implemented for use in standard care within the same patient population [10]. MHMW was built using Qualtrics software [12].

The MHMW platform was adapted for use in this study by incorporating the PRO-CTCAE (v1.0) [7] survey along with date functionality; with the psychosocial components of the MHMW platform remaining within the build [10]. Ten items from the English version of the PRO-CTCAE [13] were incorporated to the platform: nausea, vomiting, constipation, diarrhoea, dyspnoea, rash, paraesthesia, concentration, pain, and fatigue along with corresponding patient assessments of presence/absence, severity, frequency, and interference in daily life. These items were chosen following surveying of senior trials staff from the haematology and medical oncology teams to identify the ten most common symptoms experienced by participants enrolling in clinical trials across the two disciplines. Composite scoring of patient assessments of severity, frequency, and interference as reported in Basch et al. [14] was automated in the background of the platform to provide a single severity score for each reported symptom. Date functionality was added to the platform which allowed participants to enter dates for symptom onset and resolution; the platform tracked symptoms and prompted participants to confirm if previously-reported symptoms were ongoing. Free text fields were available for participants to add additional symptoms not included in the above list.

Patients were enrolled in the study once they completed screening assessments for their clinical trial and were found eligible to continue to treatment. At this time, participants were orientated to the platform by members of the research team to complete a profile (basic demographic information) and a baseline symptom survey prior to treatment start. Recall period for reporting was seven days. Survey links were automated to be sent to participants via SMS or email on a weekly basis per participant preference, with reminders sent automatically after 24 h if the survey was not completed. If the survey was still not completed, a member of the research team contacted the participant to request completion of the survey. As the platform is web-based, participants were able to complete the survey at a convenient time and location.

### Data Collection

A paper-based implementation survey adopted from Weiner et al. [15] was delivered to participants at enrolment to the study, at the end of week 12 of treatment, and at the end of week 26 of treatment. This survey rated a participant’s perception on 3 domains assessing the acceptability, appropriateness, and feasibility of the intervention measure. Each domain (acceptability of intervention measure (AIM), intervention appropriateness measure (IAM), feasibility of intervention measure (FIM)) consists of four items rated on a 5-point Likert scale (strongly disagree to strongly agree). The on-study and end-of-study versions of the implementation survey also included an item to assess participants’ perception on the time taken to complete the MHMW survey. This was in the form of a 5-point Likert scale (1 = far too little; 2 = too little; 3 = just the right amount of; 4 = too much; 5 = far too much).

To further explore participant experiences of using the MHMW platform a face-to-face, semi-structured interview was conducted at the end of study participation. The interview explored participants’ perceptions relating to the survey platform, if/how the survey impacted doctor-patient interaction, and future considerations for the survey platform. All interviews were audio-recorded and transcribed verbatim.

### Analysis

Participant demographic data was analysed using IBM SPSS Statistics [16] using descriptive statistics.

Weiner et al. [15] do not provide instruction on analysis of the implementation survey. Therefore, our analysis followed previous studies which either calculated means from individual item scores across a domain (item score) [17] or summed scores for each domain and then averaged scores across participants to obtain a single score (overall score) [18]. This results in a final score for each domain of 1 to 5 (item score) or 4 to 20 (overall score). Higher scores reflect greater acceptability, appropriateness, and feasibility. Scores ≥4 (item score) or ≥16 (overall score) reflected agree or strongly agree outcomes. Implementation survey responses were imported to IBM SPSS Statistics and means and standard deviations were calculated for each domain at each timepoint. Change over time was assessed by collapsing responses into agree/strongly agree; neutral; and disagree/strongly disagree groups then calculated using Fisher’s exact test.

Transcripts of the interviews were analysed thematically in NVivo [19] using an inductive approach to derive meaningful units per Braun and Clarke [20].

## Results

### Characteristics of Study Participants

A total of 18 patients were consented to the study; four patients were excluded from analysis due to non-compliance, having completed no MHMW surveys after the baseline survey. One of these four participants was unable to complete further surveys due to technical issues, the other three participants did not provide a reason and were lost to participation. Demographic data for the remaining cohort (*n* = 14) is described in Table 1. Average age at consent was not significantly different between the genders (*p* = 0.165) or chosen mode of delivery (*p* = 0.073).

**Table 1:** Patient demographic data

Characteristic	Number (SD, range) Total *n* = 14	%
Gender: Male Female Other	7 7 0	50 50 0
Age at consent (years): All participants Male Female	60.5 (19.9, 19.8–81.5) 67.1 (18.9, 32.2–81.5) 54.0 (20.1, 19.8–76.6)	
Preference for survey delivery: SMS Email	7 7	50 50
Time on study: Full study period completed Full study period not completed	8 6	57.1 42.9
Reason for not completing study period: Death Discontinued clinical trial Patient decision Technology issues	3 1 1 1	21.4 7.1 7.1 7.1

### Implementation Survey

Calculated means for the domains of the implementation survey over the life of the study can be found in Table 2.

**Table 2:** Mean scores for AIM, IAM, and FIM domains on the implementation survey

	Baseline			On-study			End-of-study		
	AIM	IAM	FIM	AIM	IAM	FIM	AIM	IAM	FIM
Item scores mean (SD)	3.84 (0.81)	3.80 (1.09)	3.84 (1.07)	4.08 (0.69)	4.32 (0.67)	4.18 (0.82)	4.31 (1.10)	4.25 (1.16)	4.44 (0.73)
Overall scores mean (SD)	15.36 (3.23)	15.18 (4.36)	15.36 (4.27)	16.30 (2.75)	17.27 (2.69)	16.73 (3.29)	17.25 (4.40)	17.00 (4.66)	17.75 (2.92)
Time mean (SD)				3.18 (0.40)			3.00 (0.54)		

Abbreviations: AIM-acceptability of intervention measure; IAM-intervention appropriateness measure; FIM-feasibility of intervention measure [15]

At baseline, participants scored the acceptability, appropriateness, and feasibility of the platform with a high neutral score. At the on-study timepoint the scores improved slightly to agreement. End-of-study scores remained relatively stable at the agree level. The time taken to complete the survey was rated by participants at the on-study and end-of-study timepoints with a score that corresponds to the survey taking “just the right amount” of time to complete.

With respect to change in participants’ perceptions over time, Fisher’s exact tests performed on each survey item yielded no significant change on item scores (Table 3).

**Table 3:** Fisher’s exact test of change of perceptions over time

	Baseline			On-study			End-of-study			Fisher’s
	SA + A	N	D + SD	SA + A	N	D + SD	SA + A	N	D + SD	
AIM1	9	2	0	9	2	0	7	1	0	0.999
AIM2	7	3	1	7	2	1	6	0	2	0.644
AIM3	7	4	0	6	4	0	6	1	1	0.484
AIM4	9	1	1	8	2	0	6	1	1	0.906
IAM1	8	2	1	10	1	0	6	1	1	0.788
IAM2	9	1	1	10	1	0	6	1	1	0.932
IAM3	9	1	1	10	1	0	6	1	1	0.932
IAM4	8	2	1	9	2	0	6	1	1	0.954
FIM1	9	1	1	8	3	0	7	1	0	0.649
FIM2	9	1	1	8	3	0	7	1	0	0.649
FIM3	9	1	1	8	3	0	7	1	0	0.649
FIM4	9	1	1	9	2	0	6	2	0	0.878
Time				2	9	0	1	6	1	0.745

Abbreviations: SA-strongly agree; A-agree; N-neutral, D-disagree; SD-strongly disagree

### Qualitative Interview

Perceptions of the survey platform were explored qualitatively for the eight participants that completed the study period. Three major themes emerged from the qualitative interview data: accessibility and useability, platform functionality, and personal attributes and benefits/burdens; exemplar quotes for each theme can be found from Table 4.

**Table 4:** Participant quotes extracted from interview transcripts

Theme	Participant quote
Accessibility and useability	1. “It’s just the fact that it’s easy to use basically, just get on the phone and you can just use it straight away, so it’s a good thing.” (H002) 2. “Well, I liked that it was on your phone, so it was, for me, easily accessible.” (M007) 3. “I enjoyed that it came on email and I could do it really quickly, it didn’t take long.” (M001)
Platform functionality	4. “… if you had a new symptom… when did it start? If you did it this week and said it started this week, if you didn’t keep a note, you’d forgotten when it started again by the time they asked you the following week. It was just keeping track of dates.” (M001) 5. “Kind of hard to track because, yeah, actually I don’t just track well when did it actually stop and put the date on it. So that got a bit annoying, I would say.” (H002) 6. “… I wasn’t depressed; just lethargic. On the questionnaire, there wasn’t really a place to put that. I suppose I could have written it in later.… We’re basically lazy. It’s just easier to press a button, you know?” (M009) 7. “Well, it’s so short you could actually do it more often and be more accurate, I think. Yeah. It really wouldn’t hurt to do it each morning would it, because it really only takes minutes.” (M001) 8. “I think for myself it would be better… when you have the symptoms– if you had an application…to go in there and say ‘yeah today I’m feeling– I have these symptoms.’ And when it stops being able– ‘well I feel better today’ so you have track of that already.… having more of a real time kind of thing going on…” (H002) 9. “I used to try and leave it till, say, Thursday because I have treatment of a Wednesday. So ideally I’d do it of a Thursday.” (M009) 10. “… it also reminded me to think about the things when I was coming into the clinic the next day.” (H003) 11. “I’m just wondering if those categories maybe could be a little bit more detailed” (M007)
Personal attributes and benefits/burdens	12. “I liked being able to report between my normal appointments and visits… I’d think, oh good, maybe I can let someone know about this extra bit of pain that I have here, or whatever. So that was a good thing to have that in between appointments.” (H004) 13. “I’ve been in certain moods with the treatment; ups and downs. I’ve had so much going on. Every now and then it would pop up, just at an inconvenient time where I’d just think, oh, I don’t have time right now to do this.” (H004) 14. “I won’t say I was the best at it…it had to be done… it wouldn’t be worth it if I wasn’t doing it 100 per cent. I wouldn’t benefit from it. So I suppose just knowing that it was something that was there to help me.” (H004) 15. “…if you’re in the middle of treatment you don’t really feel good–you don’t want to spend time explaining what’s wrong. Even if it’s not that long…” (H002) 16. “Depending on what the issue was. I was fine with the platform but if I had anything medically I needed to ask then I would go straight to the doctor.” (H007) 17. “I don’t have a preference either way, I do like face to face, but if the doctor’s getting the information on a survey it’s either/or, as long as he’s getting the information and making the right decisions for me.” (H003) 18. “Made me think about some of the topics, yes.” (H003) 19. “Well, by writing them down actually helped you remember to tell the doctor. It did, because otherwise it’s just all in your head and you do forget. When you go into a doctor, you become blank anyway, don’t you? Well, I do.” (M001)
Future considerations	20. “… as long as they’re well enough to answer it, to do it” (H004) 21. “So it’s definitely worthwhile. It’s definitely worthwhile.” (M007) 22. “Yes I think it is and that’s the reason why I did it, I thought it must help other people.” (H003)

#### Theme 1: Accessibility and Useability

This theme related to how participants reported they engaged with MHMW as a means to report their symptoms. All eight participants reported that the platform was easy to use, which was also identified as an enabler to completing the survey (quote 1). Participants also liked that the survey was quick and straight-forward, and was easily accessible on personal devices and able to be done from any convenient location (quotes 2,3).

#### Theme 2: Platform Functionality

Primarily, dislikes centred around platform functionality, with two participants disliking inputting dates into the platform, citing concerns around accuracy of recall from earlier in the week (quotes 4,5). Other dislikes included the interface of the platform, ambiguity in phrasing of what constituted the reporting period including lack of direction if a survey was missed one week, and the reminders that were sent if the survey was not completed. One participant described how the symptom list did not accurately reflect their experience and that there was reluctance to describe the specific nature of their symptom in the free text field (quote 6).

Interview questions exploring platform functionality specifically addressed patient perceptions on the frequency of survey completion. All eight participants agreed that weekly was an appropriate timeframe for completing the survey. Two participants further offered the view that more frequent reporting would be feasible (quotes 7,8). Timing of survey delivery was a consideration for two participants, participant M009 preferred the survey be delivered the day after treatment, while participant H003 preferred the survey be delivered the day before the clinic appointment with the doctor (quotes 9,10).

Further feedback on the functionality of the platform positively referenced the survey layout. While one participant would have liked more definitions around how to score the severity of their symptoms, three participants highlighted that the list of symptoms could be more comprehensive (quote 11).

#### Theme 3: Personal Attributes and Benefits/Burdens

Finally, participants reported that they perceived personal benefits in completing the survey, finding reassurance in knowing there was contact with the medical team outside of scheduled appointments (quote 12). Conversely, participant H004 expressed that the survey added burden to their patient journey (quote 13).

In addition to identifying ease of use as an enabler to completing the survey, participants also identified external/internal factors and treatment/disease-related factors as enablers. External/internal factors enabling completion of the survey included the use of a diary or notebooks as a reminder to complete the survey or to supplement recall. The delivery of the survey link via SMS or email along with reminders by family members, or reliance on memory also served as enablers to complete the survey. Two participants reported symptom burden and the perception that the platform was a tool there to help them as enablers (quote 14).

Five of the eight participants interviewed cited having no barriers to completing the survey. The remaining three participants cited date inputting, personal motivation relating to symptom burden, and reluctance to dwell on negative aspects of the treatment as barriers (quote 15).

Six participants valued face to face interactions with their doctor. It was important to patients that the information they entered electronically was reaching the medical team to support their clinical decision making. Participant H007 responded that preference for reporting was dependent on the issue being reported and participant H003 didn’t have a preference, so long as the information was reaching the medical team (quotes 16/17). Five of the eight participants agreed when asked if completing the survey helped in their conversations with their doctor (quotes 18,19).

### Future Considerations

The final interview questions requested suggestions for changes to improve the platform and addressed future use of the survey platform for clinical trial participants. Suggestions for improvement reflected the dislikes, barriers, and negative feedback on platform functionality. One participant highlighted that symptom burden may impact completion of reporting electronically (quote 20). All eight participants agreed that the platform would be useful for future patients (quotes 21,22).

## Discussion

This study explored cancer clinical trial participants’ experiences and perceptions of reporting their symptomatic AE data via an electronic, web-based platform, My Health My Way (MHMW). Participants agreed that MHMW was an acceptable, appropriate, and feasible intervention, and this agreement persisted through the duration of participation on the study. Interview analysis revealed that participants found MHMW to be easy and accessible to use, provided reassurance in their treatment journey in between appointments, and helped in conversations with their doctor. Areas for improvement of MHMW centred around platform functionality and concerns around symptom burden limiting responses, but despite this, MHMW was identified as a valuable tool for future clinical trial participants.

Acceptability, appropriateness, and feasibility are outcomes that are relevant, not only to assessing implementation strategies, but are also useful in assessing stakeholder perceptions of clinical interventions [15]. Assessing stakeholder perceptions early in research provides valuable insight into ensuring interventions are optimised and meet end-user preferences [15]. In our study, participants agreed that MHMW was acceptable to them, that it was an appropriate way to report their side effects, and that the platform could be successfully used by clinical trial participants. This agreement improved slightly, though not significantly from baseline, as participants became more familiar with the use of the platform and their treatment journey progressed. These results lend evidence to other studies that have investigated the feasibility of implementing patient-reported AEs in cancer clinical trials and found high adherence rates of patients completing surveys [4, 9, 21, 22].

Kennedy et al. [23] and Bruner et al. [24] each conducted interviews with stakeholders involved in cancer clinical trials, including clinical trial participants or patient advocates involved in the development, conduct, or analysis of clinical trials. These interviews explored the views of participants around the collection of patient-reported AE data; unfortunately, the participants did not complete any actual patient-reported AE tools as a part of the studies. Despite this, the views of the patient/patient advocate participants reflect those shared by the participants in our study. Kennedy et al. [23] reported that participants were happy for patient-reported AE data to be received by their treating team and expected that the data would be acted on as part of clinical management. This is mirrored by our participants who felt reassurance in having contact with the medical team in between routine appointments. In both studies, patient/patient advocate participants were supportive of reporting side effects via an electronic patient-reported AE platform and agreed that weekly was an appropriate timeframe for completion. Interestingly, several patient participants in the Kennedy et al. [23] study wanted flexibility in reporting timeframes to allow for ad hoc reporting of new symptoms, or a purely ad hoc system. This is reflected in the feedback obtained in our study, where two participants shared that reporting could be completed more frequently or on an as-needed basis.

Symptom burden as a barrier to completing patient-reported AEs was another common theme between our participants and Kennedy et al. [23] and Bruner et al. [24]. Two patient participants in Kennedy et al. [23] discussed that they felt they would only be able to report when feeling well. This is a concern shared by two of our participants. One participant, when asked if the survey would be a useful or effective tool for future cancer clinical trial participants responded affirmatively with the qualifier “*as long as they’re well enough to answer it*” (H004). The second participant, when asked if they experienced any barriers to completing the survey responded with a comment stating that when not feeling well, they “*[didn’t] want to spend time explaining what’s wrong*” (H002). When prompted by the interviewer, the participant confirmed with a sentiment that when there is already a lot going on they did not want to dwell on the negatives when not feeling well during treatment. Bruner et al. [24] suggested that proxy reporting by caregivers should be considered in cases where patients were too ill to self-report. Many systems have been developed to identify deteriorating patients while inpatients, particularly in the era of the electronic medical record [25, 26]. Fewer systems exist for monitoring deterioration in outpatients, however wearable devices show potential for addressing this gap [27]. In terms of the cancer clinical trial participant, data on patient outcomes as a result of patient-reported AE data collection is lacking. Several studies, however, have investigated the impact of patient-reported AE platforms on patient outcomes in standard care [28–30]. These studies showed that incorporating patient-reported AE platforms improved health-related quality of life, resulted in fewer emergency visits and hospitalisation and longer tolerance of chemotherapy, increased overall survival, and improved accuracy of symptom assessment and control. These same benefits could reasonably be extrapolated to the cancer clinical trial participant. With the potential for improved outcomes shown when patient-reported AE platforms are implemented, managing symptom burden and providing for capacity in the platform build for caregivers to complete proxy reporting as suggested in Bruner et al. [24] should be considered.

Kennedy et al. [23] and Bruner et al. [24] also explored platform functionality with their participants. Many of the considerations raised by their participants were either included in our build or were raised by participants of the current study as potential improvements on the platform. Common themes were that platforms should be easy to use, reminders were valuable in improving adherence to reporting, concepts and terminology should be understandable and explained clearly, accessibility and ability to report AEs between appointments, and free-text fields for inputting of any additional symptoms. The additional burden experienced by clinical trial participants of completing surveys in addition to completing treatment means careful consideration should be given to survey design. As raised by participant M009, there was a reluctance to use the free-text field “*We’re basically lazy. It’s just easier to press a button…*”. A comprehensive symptom list that reflects the expected symptoms that a patient may experience on their line of treatment should be developed. This symptom list, however, should be balanced with the risk of overburdening the participant with a lengthy list that could contribute to survey fatigue. A concern raised in Bruner et al. [24] that a patient-reported AE platform may diminish communication between patients and doctors is not reflected in either our study or other feasibility studies [5, 6]. Basch et al. [5] and Basch et al. [6] both found that use of a patient-reported AE platform in the cancer clinical trial setting improved patient-doctor communication. Two studies performed in the standard care setting also reported improved patient-doctor communication when symptomatic data was reported by patients to their doctor, on paper or electronically [31, 32].

Historically, participation in clinical trials has been reported to be low [33, 34]; barriers to participating can be categorised into domains: structural (trial availability, geographical accessibility), clinical (eligibility), physician attitudinal (physician–patient relationship, treatment preference, resourcing for cost, time, clinic), and patient attitudinal [34–36]. Patient attitudinal reasons for not participating include fear surrounding clinical trials, potentially arising from mistrust in medical science due to the often-egregious history of medical research. Other reasons include concerns around toxicity of treatments, particularly in terms of experimental therapies, and the burden of participation in a cancer clinical trial. Patient monitoring during a clinical trial can often involve more frequent visits and incur greater financial costs to participants. Finally, patients may also express a fear of experimentation when citing barriers to participating in a clinical trial [35]. An interesting point raised by a participant in the interviews in Bruner et al. [24 p.112] deserves comment: *“Self-reporting of adverse symptoms… makes the patient feel a part of the research process and not just a ‘guinea pig’ which is very important*.” The term “guinea pig” is one that, colloquially, is common when referring to patient participation in research. The fact that, as shown by the quote in Bruner et al. [24], and as reported by the participants in the current study, as well as in Liu et al. [9], collection of patient-reported AEs can provide cancer clinical trial participants reassurance and a feeling they are more involved in their care. This alone justifies the inclusion of patient-reported AE platforms in cancer clinical trial design. Clinical trial participants provide an incredibly valuable contribution to the community and deserve in return to be provided with the best experience available. An improved experience for clinical trial participants can, perhaps, work to overcome some of the fears around participating in a clinical trial, however further research is clearly needed to validate this notion.

A limitation of this study is the small sample size that was investigated. A small sample size was chosen for this study in order to investigate initial feedback on the platform to inform the potential performance of the platform in our cancer clinical trial unit with our patient population. We now look forward to incorporating the feedback on platform functionality from participants in the current study into the next platform build and testing a workflow across a subset of our trials to further optimise MHMW. A separate publication of this study reports on participant adherence in completing surveys in detail and investigates the impact of the burden of work for research team members of collecting AE data via MHMW compared with the standard current process of clinical note review [37].

## Conclusion

To our knowledge, this is the first study that has explored, in-depth, the perceptions and experiences of cancer clinical trial participants reporting the side effects of their treatment via an electronic platform. This study showed that cancer clinical trial participants are willing to report their symptomatic AE data to trial staff and they believe that reporting the data is feasible and would be of benefit to future cancer clinical trial participants. The study identified areas for improvement in the platform, and factors to consider that form barriers to patients utilising patient-reported AE platforms.

## Data Availability

The data that support the findings of this study are available from the corresponding author upon reasonable request.
